# The Mode of Action of Cyclo(l-Ala-l-Pro) in Inhibiting Aflatoxin Production of *Aspergillus flavus*

**DOI:** 10.3390/toxins9070219

**Published:** 2017-07-12

**Authors:** Kurin Iimura, Tomohiro Furukawa, Toshiyoshi Yamamoto, Lumi Negishi, Michio Suzuki, Shohei Sakuda

**Affiliations:** 1 Department of Applied Biological Chemistry, The University of Tokyo, 1-1-1 Yayoi, Bunkyo-ku, Tokyo 113-8657, Japan; iimurak@fc.jwu.ac.jp (K.I); 9000163386@mail.ecc.u-tokyo.ac.jp (T.F.); ijkwxyz@gmail.com (T.Y.); amichiwo@mail.ecc.u-tokyo.ac.jp (M.S.); 2 Institute of Molecular and Cellular Biosciences, The University of Tokyo, 1-1-1 Yayoi, Bunkyo-ku, Tokyo 113-8657, Japan; lnegishi@iam.u-tokyo.ac.jp

**Keywords:** aflatoxin, glutathione *S*-transferase, inhibitor, diketopiperazine, *Aspergillus flavus*

## Abstract

Cyclo(l-Ala-l-Pro) inhibits aflatoxin production in aflatoxigenic fungi without affecting fungal growth. The mode of action of cyclo(l-Ala-l-Pro) in inhibiting aflatoxin production of *Aspergillus flavus* was investigated. A glutathione *S*-transferase (GST) of the fungus, designated AfGST, was identified as a binding protein of cyclo(l-Ala-l-Pro) in an experiment performed using cyclo(l-Ala-l-Pro)-immobilized Sepharose beads. Cyclo(l-Ala-l-Pro) specifically bound to recombinant AfGST and inhibited its GST activity. Ethacrynic acid, a known GST inhibitor, inhibited the GST activity of recombinant AfGST and aflatoxin production of the fungus. Ethacrynic acid reduced the expression level of AflR, a key regulatory protein for aflatoxin production, similar to cyclo(l-Ala-l-Pro). These results suggest that cyclo(l-Ala-l-Pro) inhibits aflatoxin production by affecting GST function in *A. flavus*, and that AfGST inhibitors are possible candidates as selective aflatoxin production inhibitors.

## 1. Introduction

Aflatoxins are potent carcinogenic toxins that are produced by some *Aspergillus* species. Aflatoxin contamination in crops occurs globally, and is associated with high health risk and severe economic losses [1,2,3]. Therefore, effective methods for the control of aflatoxin contamination are required.

Aflatoxins decompose minimally during crop cultivation, storage, and processing due to their chemical stability. Thus, aflatoxin contamination must be controlled by reducing the amount of this toxin produced in crops. Biocontrol is a possible method for reducing aflatoxin contamination in crops, and the method using atoxigenic strains has been used in practice [4]. On the other hand, antifungal agents, which can kill mycotoxin-producing fungi, are used to inhibit mycotoxin production in general. However, the effects of fungicides against aflatoxigenic fungi in fields are limited [5].

The use of aflatoxin production inhibitors is another possible approach to aflatoxin control. As aflatoxins are produced as fungal secondary metabolites, aflatoxin production inhibitors that do not affect fungal growth may be useful as selective aflatoxin control agents without incurring the rapid spread of resistant strains. Numerous aflatoxin production inhibitors have been obtained from a variety of sources, such as plants, microbes, pesticides, and food additives [6,7,8,9]. We are using selective inhibitors that we obtained as biochemical probes to investigate the regulatory mechanism of aflatoxin production in fungi, which is very important as basic research for the development of effective aflatoxin control methods. Identification of the target molecules of the inhibitors to elucidate their modes of action is a key part of this research [10].

**Figure 1:**
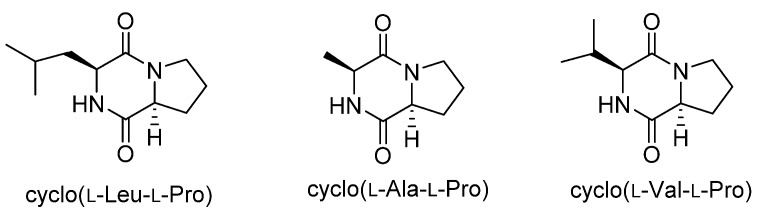
Structures of diketopiperazines with aflatoxin-production inhibitory activity.

Cyclo(l-Leu-l-Pro) was isolated from *Achromobacter xylosoxidans* as an aflatoxin production inhibitor in 2004 [11], and we recently isolated cyclo(l-Ala-l-Pro) and cyclo(l-Val-l-Pro) as aflatoxin production inhibitors from *Stenotrophomonas* sp. [12]. These diketopiperazines (Figure 1) strongly inhibited aflatoxin production in *Aspergillus flavus* and *Aspergillus parasiticus* at concentrations of a few millimolars without affecting fungal growth. Furthermore, they reduced the mRNA level of *aflR* in *A. parasiticus* [11,12]. The gene *aflR* encodes a key regulatory protein for aflatoxin production. Expression of AflR is absolutely necessary for aflatoxin biosynthesis [13], but the regulatory mechanism leading to this expression is not well understood. Therefore, studies on the mode of action of these diketopiperazines may provide an important clue to understanding the regulatory mechanism for AflR expression and aflatoxin production. In this study, we investigated the mode of action of cyclo(l-Ala-l-Pro) in inhibiting aflatoxin production through identification of its binding protein. 

## 2. Results

### 2.1. Identification of Cyclo(l-Ala-l-Pro) Binding Protein

**Figure 2:**
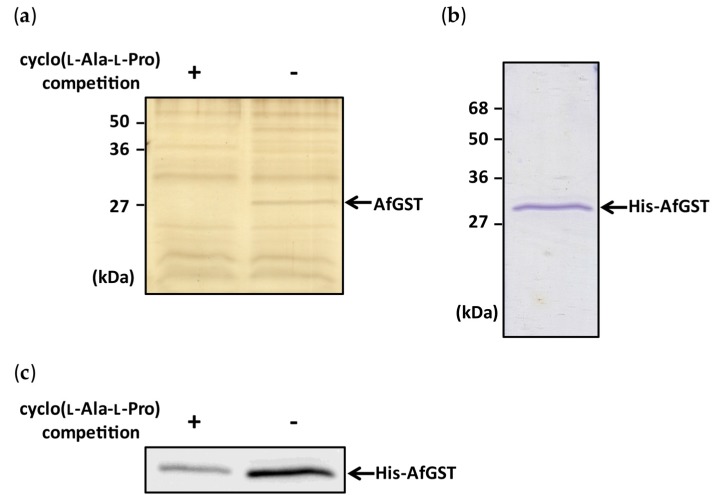
Binding experiments with cyclo(l-Ala-l-Pro)-immobilized Sepharose beads and preparation of His-AfGST. (**a**) Cyclo(l-Ala-l-Pro)-binding protein was purified from the cell lysate of *A. flavus* using cyclo(l-Ala-l-Pro)-immobilized Sepharose beads. Compared with the competitive inhibition condition (+), a strong protein band (arrow) was observed under the non-competitive inhibition condition (−). (**b**) Recombinant His-AfGST was expressed in *E. coli* and purified by a Ni Sepharose 6 Fast Flow affinity resin column. (**c**) His-AfGST was incubated with cyclo(l-Ala-l-Pro)-immobilized Sepharose beads. His-AfGST bound to the beads was detected with anti-His antibody. Band intensity observed under the non-competitive inhibition condition (−) was greater than that observed under the competitive inhibition condition (+).

To purify a binding protein of cyclo(l-Ala-l-Pro) by an affinity bead method, cyclo(l-Ala-l-Pro)-immobilized Sepharose beads, designated LL-beads, were prepared through a cross-linking reaction between the cyclo(l-Ala-l-Pro) molecule and the diazirine group of 4-[3-(trifluoromethyl)-3*H*-diazirin-3-yl]benzoic acid [14]. LL-beads were incubated with the cell lysate of *Aspergillus flavus* IMF 47798 and collected. Bead-binding proteins were eluted from the beads with a sodium dodecyl sulfate-polyacrylamide gel electrophoresis (SDS-PAGE) sample buffer and the eluate was analyzed by SDS-PAGE. Many bands were detected on the resulting gel (Figure 2a), but a band around 27 kDa disappeared clearly when cyclo(l-Ala-l-Pro) was added to the protein extracts before incubation with LL-beads (left lane in Figure 2a), suggesting specific binding of cyclo(l-Ala-l-Pro) to a protein involved in the 27 kDa band. Protein in the band was digested with trypsin and subjected to liquid chromatography/tandem mass spectrometry (LC/MS/MS) analysis. The highest-scoring candidate protein in this analysis (Table S1) was *A. flavus* glutathione *S*-transferase (XP_002372565), designated AfGST. 

A pull-down experiment with a recombinant protein was performed to confirm the binding of cyclo(l-Ala-l-Pro) to AfGST. His-tagged recombinant AfGST (His-AfGST) was bacterially expressed and purified (Figure 2b). His-AfGST bound clearly to LL-beads, as shown by the same assay depicted in Figure 2a, and the intensity of the band became very weak when cyclo(l-Ala-l-Pro) was mixed with His-AfGST before incubation with LL-beads (Figure 2c). These results indicated the specific binding of cyclo(l-Ala-l-Pro) to AfGST.

The amino acid sequence of AfGST showed homology to *Aspergillus fumigatus* GstA (AAX07320), *A. fumigatus* GstB (AAX07318), *A. fumigatus* GstC (AAX07319), and *A. nidulans* GstA (AAM48104), at levels of 46%, 68%, 39%, and 40% identity, and 81%, 91%, 73%, and 73% similarity, respectively [15,16]. AfGST showed the greatest similarity to *A. fumigatus* GstB.

### 2.2. Time Course of AfGST Expression

**Figure 3:**
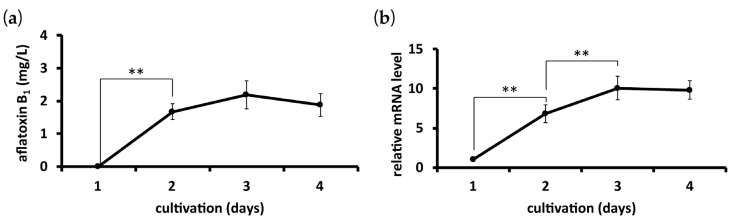
Time courses of aflatoxin B_1_ production and mRNA level of the gene encoding AfGST. Strain IMF 47798 was cultured in a PDB liquid medium for 4 days, and the time courses of aflatoxin B_1_ production (**a**) and the mRNA level of the gene encoding AfGST (**b**) were measured. Error bars show the standard deviations. *n* = 4 or 5, ** *p* < 0.01 between timepoints.

Strain IMF 47798 was cultured in a potato dextrose broth liquid (PDB) medium for 4 days. Time courses of AfGST expression and aflatoxin B_1_ production by the fungus during cultivation were measured. Aflatoxin production increased markedly from day 1 to day 2 of cultivation, and then increased gradually to reach a maximum on day 3 of cultivation (Figure 3a). Similarly, the mRNA level of the gene encoding AfGST increased markedly from day 1 to day 2 of cultivation, and then more gradually to reach a plateau (Figure 3b).

### 2.3. Effect of Cyclo(l-Ala-l-Pro) on GST Activity

**Figure 4:**
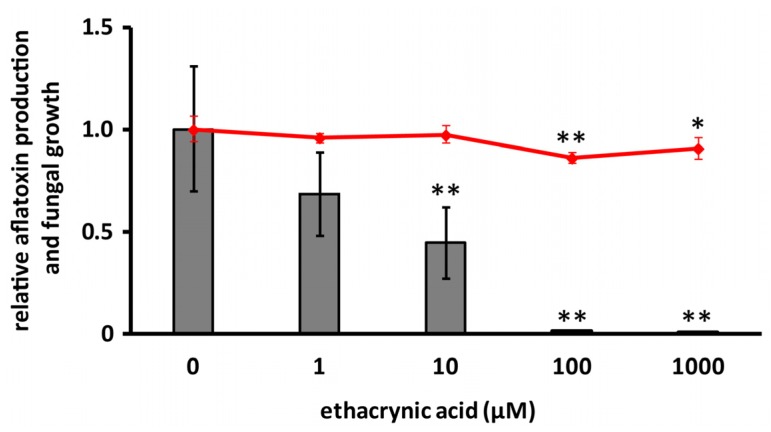
Effects of ethacrynic acid on aflatoxin production and fungal growth. The bar graph and polygonal line graph show aflatoxin B_1_ production and dry mycelial weight, respectively. Error bars show standard deviations. *n* = 3 or 4, ** *p* < 0.01, * *p* < 0.05 vs. control.

**Figure 5:**
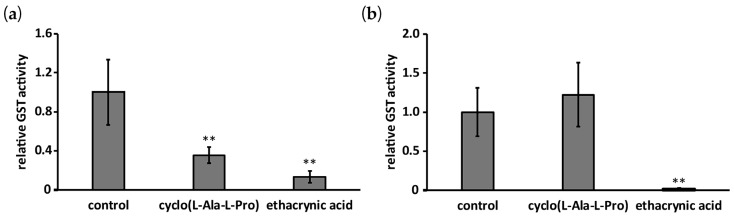
Effects of cyclo(l-Ala-l-Pro) (2 mM) and ethacrynic acid (100 μM) on GST activities of His-AfGST (**a**) and *Schistoma japonicum* GST (rShGST) (**b**). Error bars show standard deviations. *n* = 3 or 4, ** *p* < 0.01 vs. control.

His-AfGST showed glutathione *S*-transferase activity when 1-chloro-2,4-dinitrobenzene (CDNB) was used as a substrate. The effect of cyclo(l-Ala-l-Pro) on the GST activity of His-AfGST was evaluated using CDNB. Ethacrynic acid, a known GST inhibitor, was used as a positive control. Ethacrynic acid inhibited aflatoxin production of strain IMF 47798 in a dose-dependent manner (Figure 4). It inhibited aflatoxin production at the concentration of 100 μM almost completely, but its growth-inhibitory activity was very weak at 1 mM. Ethacrynic acid reduced GST activity of His-AfGST to 13% of that of the control at the concentration of 100 µM. Cyclo(l-Ala-l-Pro) also decreased the GST activity to 35% compared with the control (Figure 5a). As cyclo(l-Ala-l-Pro) reduced the amounts of aflatoxin produced by strain IMF 47798 to 25% and 10% those of the control without the compound at the concentrations of 3 mM and 1.5 mM, respectively [12], the inhibitory activities of ethacrynic acid and cyclo(l-Ala-l-Pro) against His-AfGST seemed to be correlated with their aflatoxin production inhibitory activities.

Ethacrynic acid strongly inhibited the GST activity of recombinant *Schistoma japonicum* GST (rShGST) at the concentration of 100 µM when CDNB was used as substrate. However, cyclo(l-Ala-l-Pro) did not affect the GST activity of rShGST at the concentration of 2 mM (Figure 5b).

### 2.4. Effects of Cyclo(l-Ala-l-Pro) and Ethacrynic Acid on the mRNA Level of *aflR*

**Figure 6:**
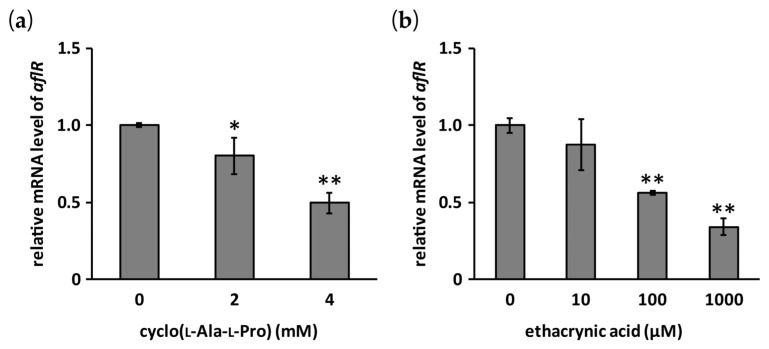
Effects of cyclo(l-Ala-l-Pro) (**a**) and ethacrynic acid (**b**) on the mRNA level of *aflR*. Error bars show standard deviations. *n* = 3 (**b**) or 4 (**a**), * *p* < 0.05, ** *p* < 0.01, vs. control.

Effects of cyclo(l-Ala-l-Pro) and ethacrynic acid on the mRNA level of *aflR* in *A. flavus* IMF 47798 were evaluated by RT-qPCR. Both compounds significantly reduced this mRNA level in a dose-dependent manner (Figure 6). 

## 3. Discussion

Cyclo(l-Ala-l-Pro), a simple diketopiperazine produced by a bacterium, inhibits aflatoxin production without affecting the growth of aflatoxigenic fungi. In this paper, we investigated the mode of action of cyclo(l-Ala-l-Pro) to begin to clarify the regulatory mechanism of aflatoxin production, which is very important for aflatoxin control.

Crosslinking using diazirine chemistry, in which reactive carbene forms a covalent bond at a random position of the cyclo(l-Ala-l-Pro) molecule, was used to prepare cyclo(l-Ala-l-Pro)-immobilized affinity beads to purify a binding protein because we could not obtain cyclo(l-Ala-l-Pro) derivatives maintaining suitable aflatoxin production inhibitory activity for the design of an affinity probe. As a result of the bead experiments, we identified AfGST as a binding protein of cyclo(l-Ala-l-Pro) by the experiments with the beads. As no GST protein from *A. flavus* has been characterized, this work is the first to examine an *A. flavus* GST protein.

GST is a detoxification enzyme. It generally catalyzes the conjugation of glutathione (GSH) with xenobiotics and endogenous products of oxidative stress [17]. Amino acid sequences of fungal GSTs do not fit the GST classes of higher eukaryotes [16]. To date, a limited number of *Aspergillus* GSTs have been studied, namely two GSTs of *A. nidulans* (GstA and GstB) [16,18] and three GSTs of *A. fumigatus* (GstA, GstB and GstC) [15]. These GSTs have been speculated to play roles in response to xenobiotics (*A. nidulans* GstA, and *A. fumigatus* GstA, GstB, and GstC), oxidative stress (*A. nidulans* GstB, and *A. fumigatus* GstA and GstC), and heavy metals (*A. nidulans* GstA). As AfGST shows great similarity to *A. nidulans* GstA, *A. fumigatus* GstA, *A. fumigatus* GstB, and *A. fumigatus* GstC, it may play a role in the response to xenobiotics and/or oxidative stress.

Saxena et al. [19,20] showed that *A. flavus* aflatoxin production and cytosolic GST activity measured using CDNB as a substrate were correlated positively, and that tolnaftate, a GST inhibitor, suppressed aflatoxin production of the fungus. This correlation was confirmed by an experiment conducted with several toxigenic and non-toxigenic isolates of *A. parasiticus* and *A. flavus* [21]. In this study, we showed that AfGST expression synchronized aflatoxin production in *A. flavus* IMF 47798 and that two AfGST inhibitors, cyclo(l-Ala-l-Pro) and ethacrynic acid, suppressed aflatoxin production of the fungus. Furthermore, cyclo(l-Ala-l-Pro) and ethacrynic acid significantly reduced the mRNA level of *aflR* dose-dependently at the approximate concentrations at which aflatoxin production was inhibited. These results suggest that GST activity plays an important role in the regulatory mechanism of aflatoxin production. Oxidative stress is thought to be a key factor for aflatoxin production of aflatoxigenic fungi [22]. For example, deletion of *veA*, encoding a transcription factor which responds to oxidative stress, is known to reduce not only transcription of oxidative stress responding genes, such as catalase and thioredoxin reductase genes, but also aflatoxin production [23,24]. AfGST may thus play a role in response to intracellular oxidative stress.

GST inhibitors are useful as therapeutic agents [25] and many GST inhibitors are known [26]. As AfGST is a possible target for the control of aflatoxin production, aflatoxin production inhibitors might be obtained from GST inhibitors and compounds identified by conducting a screening search for AfGST inhibitors. Work to investigate the function of AfGST in aflatoxin production is now in progress. 

## 4. Conclusions

Glutathione *S*-transferase (AfGST) of *A. flavus* was identified as the specific binding protein of cyclo(l-Ala-l-Pro), a selective aflatoxin production inhibitor. Cyclo(l-Ala-l-Pro) inhibited GST activity of AfGST. Ethacrynic acid, a known GST inhibitor, inhibited AfGST and aflatoxin production by reducing AflR expression, similar to the action of cyclo(l-Ala-l-Pro). AfGST was clarified as a possible target for aflatoxin control. 

## 5. Materials and Methods

### 5.1. Chemicals

Aflatoxin standard was purchased from Sigma-Aldrich (St. Louis, MO, USA). All other chemicals and solvents were purchased from Sigma-Aldrich, Kanto Chemical (Tokyo, Japan), Tokyo Chemical Industry (Tokyo, Japan), and Nacalai Tesque (Kyoto, Japan), unless otherwise specified. Cyclo(l-Ala-l-Pro) was synthesized according to the method reported previously [12]. 

### 5.2. Strains and Culture Conditions 

*A. flavus* IMF 47798 was used as a producer of aflatoxin B_1_ throughout the study. Aflatoxin B_1_ is the main aflatoxin produced by this strain. A preserved glycerol stock of a spore suspension prepared from a 1-week-old culture was used as the inoculum. The spore suspension was inoculated into PDB liquid medium in 12-well microplates (2 mL/well). Each microplate was inoculated with spore suspension of the strain (10^3^ spores/mL) and incubated at 28 °C for 24−96 h. Test compounds were dissolved in dimethyl sulfoxide and added to the wells (final concentration of dimethyl sulfoxide, 0.25% *v/v*). The plates were incubated undisturbed at 28 °C for four days.

### 5.3. Analyses of Aflatoxin Production and Fungal Growth

After four days of incubation, the culture broth of *A. flavus* IMF 47798 from each well was centrifuged to obtain the mycelia and culture supernatant. The mycelial weight and aflatoxin B_1_ concentration in the culture supernatant were analyzed according to the method reported previously [9].

### 5.4. Preparation of Cyclo(l-Ala-l-Pro)-Immobilized Sepharose Beads 

Cyclo(l-Ala-l-Pro)-immobilized Sepharose beads, named LL-beads, were prepared according to the method of Takayama et al. [27] with some modifications. A methanol solution (1 mL) containing cyclo(l-Ala-l-Pro) (6.8 mg, 40 mmol) and 4-[3-(trifluoromethyl)-3*H*-diazirin-3-yl]benzoic acid (4.6 mg, 20 mmol) was placed in a glass sample vial. After the removal of methanol from the solution *in vacuo* in the dark, the residue was irradiated at 365 nm (4 J/cm^2^) for cross-linking. The irradiated residue was mixed with a dimethylformamide (DMF) solution (1 mL) of *N*-hydroxysuccinimide (20 mmol) and 1-(3-dimethylaminopropyl)-3-ethylcarbodiimide hydrochloride (20 mmol), incubated for 2 h at room temperature, and added to Sepharose beads (1 mL, EAH Sepharose 4B; GE Healthcare, Little Chalfont, UK), which had been washed three times with 1 mL DMF before use. The mixture was incubated overnight at room temperature and the resulting beads were washed with 0.5 mL DMF three times and 0.5 mL distilled water three times successively. The beads were suspended in phosphate-buffered saline (PBS; 1 mL) and stored at 4 °C. 

### 5.5. Detection of Cyclo(l-Ala-l-Pro)-Binding Protein

The protocol reported by Kawatani et al. [14] was used as a reference. A culture flask (500 mL) containing 200 mL PDB medium was inoculated with spore suspension of *A. flavus* IMF 47798 (10^5^ spores/mL) and incubated statically at 28 °C for 24 h. The culture broth was filtered to obtain the mycelia. After washing with distilled water several times, the mycelia were suspended in 2 mL binding buffer (50 mM Tris-HCl (pH 8.0), 150 mM NaCl, 5 mM MgCl_2_, 1 mM EDTA, 0.2% Nonidet P-40, 5% glycerol, protease inhibitor mixture (Roche, Basel, Switzerland)) and homogenized manually. After the homogenates were centrifuged, the supernatant was collected as a cell lysate. The protein concentration was determined with the Bradford protein assay.

After incubation of the cell lysate (2 mg protein, 1 mL) in the presence or absence of 59.5 mM cyclo(l-Ala-l-Pro) for 2 h at 4 °C, the solution was reacted with 100 μL of LL-beads for 4 h at 4 °C. The reacted beads were collected by centrifugation and washed four times with 0.5 mL binding buffer, and bounded proteins were eluted with 50 μL SDS-PAGE sample buffer. The proteins were separated by SDS-PAGE and stained with the SilverQuest Silver Staining Kit (Invitrogen, Waltham, MA, USA). To identify the protein, the protein band on SDS-PAGE output was analyzed by LC/MS/MS according to the protocol reported previously [9]. Data processing was performed by Proteome Discoverer 1.4 (Thermo Fisher Scientific, Waltham, MA, USA) and protein data of *Aspergillus flavus* NRRL 3357 was obtained from the web site [28].

### 5.6. Binding Assay with Recombinant AfGST

Recombinant AfGST containing His_6_ at the N-terminus (His-AfGST) was prepared. *A. flavus* was cultured in PDB medium (2 mL) in 12-well microplates at 28 °C. The obtained mycelia were freeze dried and used for RNA extraction. Total RNA was extracted with TRIzol reagent (Invitrogen, Waltham, MA, USA) and PureLink RNA Mini Kit (Ambion, Waltham, MA, USA), according to the manufacturer’s protocol. The cDNA was prepared with ReverTra Ace qPCR RT Master Mix (TOYOBO, Osaka, Japan) according to the manufacturer’s protocol. PCR was performed with cDNA serving as a template using the following primers: 5′-GTCGACGTCTCTTAAGCCTATTATCCTCCATGGG-3′ and 5′-GGGAAGCTTTCAAGGATGCGTCATTTTCGAGGT-3′. PCR products were cloned into pGEM-T (Easy) Vectors (Promega, Fitchburg, WI, USA).

After digestion with HindIII and SalI, the inserts were ligated into pPRO EX HTb expression plasmid (Invitrogen). *Escherichia coli* BL21 (DE3) cells were transformed with the pPRO based expression plasmid. Transformed cells were cultured in 200 mL Lysogeny broth containing ampicillin (50 μg/mL) and grown to a final OD_600_ of 0.4 at 37 °C. Isopropyl β-d-1-thiogalactopyranoside (IPTG; 1 mM) was added to the culture, which was then incubated for an additional 16 h at 16 °C. 

Bacterial cells were collected by centrifugation and resuspended in PBS containing 0.1% Triton X100 and protease inhibitor mixture. After the cells had been disrupted by sonication and centrifuged, recombinant protein in the soluble fraction was purified with a Ni Sepharose 6 Fast Flow affinity resin column (GE Healthcare, Little Chalfont, UK), according to the manufacturer’s protocol. Recombinant AfGST was stored as 20% glycerol solution at −80 °C.

After incubating 2 µg His-AfGST in 200 µL binding buffer with or without 2 mg cyclo(l-Ala-l-Pro) (59.5 mM) for 2 h at 4 °C, the solution was incubated with 20 µL LL-beads for another 3 h at 4 °C. The beads were washed four times with 200 µL binding buffer and the bound proteins were separated as described in Section 5.5. The protein was detected by Western blotting, as reported previously [10], using secondary peroxidase-conjugated goat polyclonal anti-mouse IgG (H+L) antibody (Thermo Fisher Scientific, Waltham, MA, USA) and ECL Prime Western Blotting Detection Reagent (GE Healthcare, Little Chalfont, UK).

### 5.7. RT-qPCR Analysis

The cDNA derived from 12.5 ng total RNA was used as a template. qPCR was carried out using FastStart Universal SYBR Green Master (Rox) (Roche) in a final volume of 15 μL for each reaction and an ABI PRISM 7300 thermal cycler (Applied Biosystems, Waltham, MA, USA). Two-step PCR conditions were as follows: initial incubation at 95 °C for 10 min, followed by 40 cycles at 95 °C for 15 s and 60 °C for 1 min. The expression was normalized to the amount of β-tubulin expression in each sample. The following PCR primers were used for the genes: β-tubulin, 5′-AGCTCTCCAACCCCTCTTACG-3′ and 5′-TGAGCTGACCGGGGAAACG-3′, *AfGST*, 5′-GCGCTACGTCAACGAAATCC-3′ and 5′-TGCGTCATTTTCGAGGTTGC-3′, *aflR*, 5′-TCCGCCATCTTTTCTCATCA-3′ and 5′-CCGAATTCCGAATCGACTGTTA-3′.

### 5.8. GST Activity of His-AfGST and Schistoma Japonicum GST

GST activity was measured according to the method described by Habdous et al. [29] with some modifications. The His-AfGST (0.5 μg) in 30 μL of 0.1 M phosphate buffer (PB) (pH 6.5) was mixed with cyclo(l-Ala-l-Pro) or ethacrynic acid and incubated at 4 °C for 1 h. After incubation, 470 μL 0.1 M PB (pH 6.5) containing CDNB and GSH was added to the mixture. The final concentration of CDNB, GSH, cyclo(l-Ala-l-Pro), and ethacrynic acid were 1 mM, 1 mM, 2 mM, and 100 μM, respectively. Enzyme activity was calculated according to the change in an absorbance at 340 nm after 5 min. A control experiment was performed without cyclo(l-Ala-l-Pro) or ethacrynic acid. 

The vector used to express GST-tagged fusion protein (pGEX-6P-1) was used to prepare rShGST. *E. coli* BL21 (DE3) cells containing pGEX-6P-1 were cultured as described in Section 5.5, and rShGST was purified with a Glutathione Sepharose 4 Fast Flow column (GE Healthcare, Little Chalfont, UK) according to the manufacturer’s protocol. rShGST was stored as 20% glycerol solution at −80 °C.

### 5.9. Statistical Analysis 

Data are presented as means and standard deviations. Differences among the three groups were assessed by one-way analysis of variance, followed by the Tukey-Kramer test or Dunnett test. *p* values < 0.05 were considered to be significant.

## Supplementary Materials

The following are available online at www.mdpi.com/2072-6651/9/7/219/s1, Table S1: Proposed proteins as cyclo(l-Ala-l-Pro)-binding protein from peptide sequences determined by LC/MS/MS.
