# Supplementary material for: The Mode of Action of Cyclo(l-Ala-l-Pro) in Inhibiting Aflatoxin Production of Aspergillus flavus
Source: Toxins (Basel). 2017 Jul 12;9(7):219. doi: 10.3390/toxins9070219 (PMC5535166; doi:10.3390/toxins9070219)
Supplement: Supplementary file 1 [file toxins-09-00219-s001.pdf]

Supplementary Materials: The Mode of Action of Cyclo(L-Ala-L-Pro) in Inhibiting Aflatoxin Production of *Aspergillus flavus*

Kurin Iimura, Tomohiro Furukawa, Toshiyoshi Yamamoto, Lumi Negishi, Michio Suzuki and Shohei Sakuda

Table S1. Proposed proteins as cyclo(L-Ala-L-Pro)-binding protein from peptide sequences determined by LC/MS/MS.

No.	Accession	Description	Score	Coverage	MW [kDa]	pI	Obtained peptides	Peptide confidence
1	XP_002372565	glutathione *S*-transferase	39.59	45.05%	25.6	6.65	VSETmNNVLADR	High
							VAMLLNELNVPYEYK	High
							FSFEAGTPEYFHAK	High
							VSETMNNVLADR	High
							RVSETMNNVLADR	High
							RVSETMNNVLADREYLVGDK	High
							VSETMNNVLADREYLVGDK	High
							YHPEKVESAR	High
							YLQFAEIK	Low
							VSETmNNVLADREYLVGDK (M5(Oxidation))	Low
							ALAEKAEATSK	Low
							YVNEIR	Low
							KFSFEAGTPEYFHAK	Low
							RVSETmNNVLADREYLVGDK (M6(Oxidation))	Low
							RVSETmNNVLADR (M6(Oxidation))	Low
							YVNEIRR	Low
							ARPAIAK	Low
							EYLVGDK	Low
							SEPFFK	Low
							IKARPAIAKALAEK	Low
2	XP_002372769	ATP synthase subunit 4, mitochondrial precursor	24.32	33.33%	26.4	9.07	ETAQLEAQAYELEQR	High

							VLQQILQQSVADVER	High
							MDNVQELAGVVEVTK	High
							mDNVQELAGVVEVTK (M1(Oxidation))	High
							ELAESVIGK	Low
							TALAHEAK	Low
							QALDSWVR	Low
							IQKELENPK	Low
							SRmDNVQELAGVVEVTK (M3(Oxidation))	Low
3	XP_001821105	hypothetical protein similar to elongation factor-1 gamma	8.99	20.09%	24.6	6.23	IQAAGNLNSLSITTSPDFQMGVTNR	High
							IQAAGNLNSLSITTSPDFQmGVTNR (M20(Oxidation))	High
							TIESEGVK	Low
							YTAEELEEHLAK	Low
4	XP_001826698	hypothetical protein AOR_1_162034	8.85	17.36%	26.3	5.15	AFEDLGYEAVcPR (C11(Carbamidomethyl))	High
							TLADDVALIR	High
							QTAQSLIDDGK	Low
							LVLKIASQ	Low
5	XP_001825196	60S ribosomal protein L1	8.05	26.73%	24.2	9.83	QNIQQLLDYSQNEK	High
							KYDAFLASDGLIK	High
							FPTPISHAEDMANK	Low
							LLGPGLSK	Low
							DKRFSGtIK (T7(Phoshorylation))	Low
6	XP_001825192	hypothetical protein similar to SURF-4 protein	3.41	7.59%	34.1	9.23	ADATSDPSPLDAIR	High
							KMyVQFAGR (Y3(Phosphorylation))	Low
7	XP_002382442	hypothetical protein similar to integral membrane protein 25D9-6	1.76	4.94%	29.9	10.24	GQIQGQFQEEAAK	High

8	XP_002374455	60S ribosomal protein L20	1.67	6.32%	20.4	10.43	VVEVDNADSIR	High
9	XP_002373130	conserved hypothetical protein	0.00	3.39%	199.9	5.86	KKNTmGTPyGcWLK (M5(Oxidation); Y9(Phosphorylation); C11(Carbamidomethyl))	High
							DVASITPsNR (S8(Phosphorylation))	Low
							LAsPTVQNR (S3(Phosphorylation))	Low
							RIMINNFSAAGGNSsVLIEDAPVFEPKsK (S15(Phosphorylation); S28(Phosphorylation))	Low
10	XP_002372894	hypothetical protein similar to endothelin-converting enzyme	0.00	2.36%	71.4	5.33	EQLFFISYANWWcSK (C13(Carbamidomethyl))	High
